# The Potential Chemopreventive Effect of *Andrographis paniculata* on 1,2-Dimethylhydrazine and High-Fat-Diet-Induced Colorectal Cancer in Sprague Dawley Rats

**DOI:** 10.3390/ijms24065224

**Published:** 2023-03-09

**Authors:** Tharani Subarmaniam, Rusydatul Nabila Mahmad Rusli, Kokila Vani Perumal, Yoke Keong Yong, Siti Hadizah, Fezah Othman, Khaled Salem, Nurul Husna Shafie, Rosnani Hasham, Khoo Boon Yin, Khairul Kamilah Abdul Kadir, Hasnah Bahari, Zainul Amiruddin Zakaria

**Affiliations:** 1 Borneo Research on Algesia, Inflammation and Neurodegeneration (BRAIN) Group, Faculty of Medicine and Health Sciences, Universiti Malaysia Sabah, Jalan UMS, Kota Kinabalu 88400, Sabah, Malaysiazaz@ums.edu.my (Z.A.Z.); 2 Department of Human Anatomy, Universiti Putra Malaysia, Serdang 43400, Selangor, Malaysia; 3 Department Biomedical Sciences, Universiti Putra Malaysia, Serdang 43400, Selangor, Malaysia; 4 Department of Nutrition, Universiti Putra Malaysia, Serdang 43400, Selangor, Malaysia; 5 Laboratory of UPM-MAKNA Cancer Research, Institute of Bioscience, Universiti Putra Malaysia, Serdang 43400, Selangor, Malaysia; 6 Department of Bioprocess and Polymer Engineering, School of Chemical and Energy Engineering, Faculty of Engineering, Universiti Teknologi Malaysia, Johor Bahru 81310, Johor, Malaysia; 7 Institute for Research in Molecular Medicine (INFORMM), Universiti Sains Malaysia, Penang 11800, Penang, Malaysia; 8 Department of Innovation and Commercialization, Forest Research Institution Malaysia, Kepong 52109, Selangor, Malaysia

**Keywords:** *Andrographis paniculata*, colorectal cancer (CRC), high-fat diet (HFD), DMH, obesity, anti-cancer

## Abstract

Colorectal cancer (CRC) is responsible for a notable rise in the overall mortality rate. Obesity is found to be one of the main factors behind CRC development. *Andrographis paniculata* is a herbaceous plant famous for its medicinal properties, particularly in Southeast Asia for its anti-cancer properties. This study examines the chemopreventive impact of *A. paniculata* ethanolic extract (APEE) against a high-fat diet and 1,2-dimethylhydrazine-induced colon cancer in Sprague Dawley rats. Sprague Dawley rats were administered 1,2-dimethylhydrazine (40 mg/kg, i.p. once a week for 10 weeks) and a high-fat diet (HFD) for 20 weeks to induce colorectal cancer. APEE was administered at 125 mg/kg, 250 mg/kg, and 500 mg/kg for 20 weeks. At the end of the experiment, blood serum and organs were collected. DMH/HFD-induced rats had abnormal crypts and more aberrant crypt foci (ACF). APEE at a dose of 500 mg/kg improved the dysplastic state of the colon tissue and caused a 32% reduction in the total ACF. HFD increased adipocyte cell size, while 500 mg/kg APEE reduced it. HFD and DMH/HFD rats had elevated serum insulin and leptin levels. Moreover, UHPLC-QTOF-MS analysis revealed that APEE was rich in anti-cancer phytochemicals. This finding suggests that APEE has anti-cancer potential against HFD/DMH-induced CRC and anti-adipogenic and anti-obesity properties.

## 1. Introduction

In terms of overall mortality rates, colorectal cancer (CRC) ranks third in males and second in females globally [1]. According to the GLOBOCAN database, the mortality rate of CRC among females (9.5%) is higher compared with males (9.3%) in the year 2020 [2]. In Southeast Asia, Malaysia is ranked as having the 3rd highest incidence and mortality of CRC. Specifically, the mortality and incidence of CRC were found to be increasing more among males than females, particularly in those of Chinese ethnicity [3]. The global economic cost of CRC care approaches USD 100 billion, with medical spending alone expected to exceed USD 20 billion [4]. As a result, there is a pressing requirement for a better knowledge of the pathophysiology of colorectal cancer as well as the discovery of new therapeutic methods.

There are several factors influencing CRC development such as age, sex, smoking, poor diet, genetics, and obesity [5]. The most prevalent risk factors for colorectal cancer (CRC) development are a high-fat diet (HFD). The diet, which has a high proportion of fat, has been linked to obesity because of its tendency to promote weight gain over time [6]. Recent epidemiological research has concluded that obesity leads to rising morbidity and mortality linked with colorectal cancer [7]. This is because a high-fat diet has been connected to pro-inflammatory potential, raising the risk of CRC. The pro-inflammatory factors that associate with obesity such as adipokines (secreted by adipocytes) and cytokines will cause low-grade inflammation, which provides a favorable environment for cancer tumor growth [8]. Likewise, a previous study showed the adipose tissue secretes high levels of adipokines and cytokines, which aid in inducing CRC in high-fat-diet-fed rats [9].

Surgery, chemotherapy, and radiation are the primary conventional therapies for CRC. These therapies can also be used in combination, depending on the location and course of the disease [10]. Fluorouracil (5-FU) is a fluoropyrimidine chemotherapeutic drug that has been extensively applied to treat a variety of malignant tumors, and particularly CRC, for over 50 years [11,12]. Even though 5-FU is one of the safest chemotherapy medicines, severe side and toxic effects occur in some CRC patients [13]. The problems of conventional chemotherapies are related to their properties as anti-metabolites that disturb the formation of vital proteins, cause subsequent cell degradation, and result in long-term adverse outcomes [14]. Therefore, the urge to develop the safest therapy has drawn researchers’ attention toward medicinal plants. Recently, significant research has been conducted on medicinal plants to understand their properties to cure certain acute diseases such as cancer [15]. Among them, *Andrographis paniculata* is one of the medicinal plants that has been broadly studied for its anti-cancer properties.

*Andrographis paniculata (A. paniculata)*, often identified as “King of Bitters”, is an Acanthaceae family plant [16]. It is grown extensively in southern Asia. Throughout the years, leaves and roots have been utilized in alternative medicine systems for a variety of therapeutic purposes, as a cure for a wide range of diseases, or as a health supplement [17]. *Andrographis paniculata* has been shown to be a promising potential cure for many diseases, especially cancer. *A. paniculata* has been found to contain a variety of phytoconstituents such as labdane diterpenoids, quinic acids, flavonoids, noriridoids, and xanthones. The major constituents of *Andrographis paniculata* are andrographolide, a labnade diterpenoid that has been studied for its chemopreventive properties [17]. For example, research has provided evidence that andrographolide that is isolated from *A. paniculata* helps to slow down the spread of CRC by inducing cell apoptosis [18].

**Figure 1:**
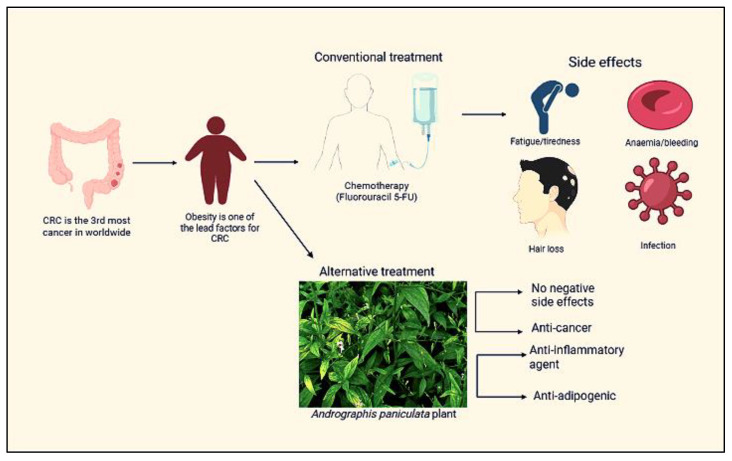
The summary of the study.

Moreover, prior research investigated the potential chemopreventive activities of *A. paniculata* against colorectal cancer. However, there is little understanding concerning the effect of *A. paniculata* on CRC under HFD conditions. This study’s goal was to examine the anti-cancer properties of *A. paniculata* ethanolic extract on 1,2-dimethylhydrazine (DMH)-induced colon cancer in Sprague Dawley rats in high-fat diet conditions (Figure 1).

## 2. Results

### 2.1. Phytochemical Screening and Identification of A. paniculata Ethanol Extract

**Figure 2:**
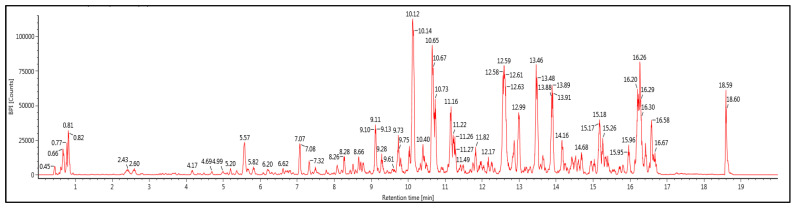
UHPLC-QTOF-MS base peak intensity (BPI) metabolic profile of ethanol extract of *A. paniculata*.

Table 1 shows a list of the main phytochemical compounds of *A. paniculata* ethanolic extract, and Figure 2 demonstrates the UHPLC-QTOF-MS base peak intensity (BPI) metabolic profile of the ethanol extract of *A. paniculata*. A total of six isolated compounds were mainly from diterpenoids, flavonoids, and quinic acid. The 19β-Glucosyl-14-deoxy-11,12-didehydroand-rographoside, 12S-Hydroxyandrographolide, 10-Hydroxyligustroside, and 19β-Glucosyl-14-deoxyandrographoside were from the terpenoids group. From flavonoids, we found the Genistein-7,4′-di-O-β-D-glucoside compound, while 3,4-O-Dicaffeoylquinic acid was from the quinic acid group.

### 2.2. Impact of A. paniculata on Food Intake, Body Weight, Retroperitoneal White Adipose Tissue (RpWAT), Colon Weight, and Colon Length

Table 2 displays the food intake and changes in the body weight, colon weight, and colon length of the treated rats. The body weight and RpWAT weight of the DMH/high-fat diet consumed by the rats were significantly lower (*p* < 0.05) than the high-fat diet given to the rats. However, there was no notable change in their food intake. Three doses of *A. paniculata* ethanolic extract had no significant effect on the body weight, RpWAT weight, colon weight, and colon length of the treated rats.

### 2.3. Impact of A. paniculata on the Histopathological Finding of the Colon in Treated Rats

**Figure 3:**
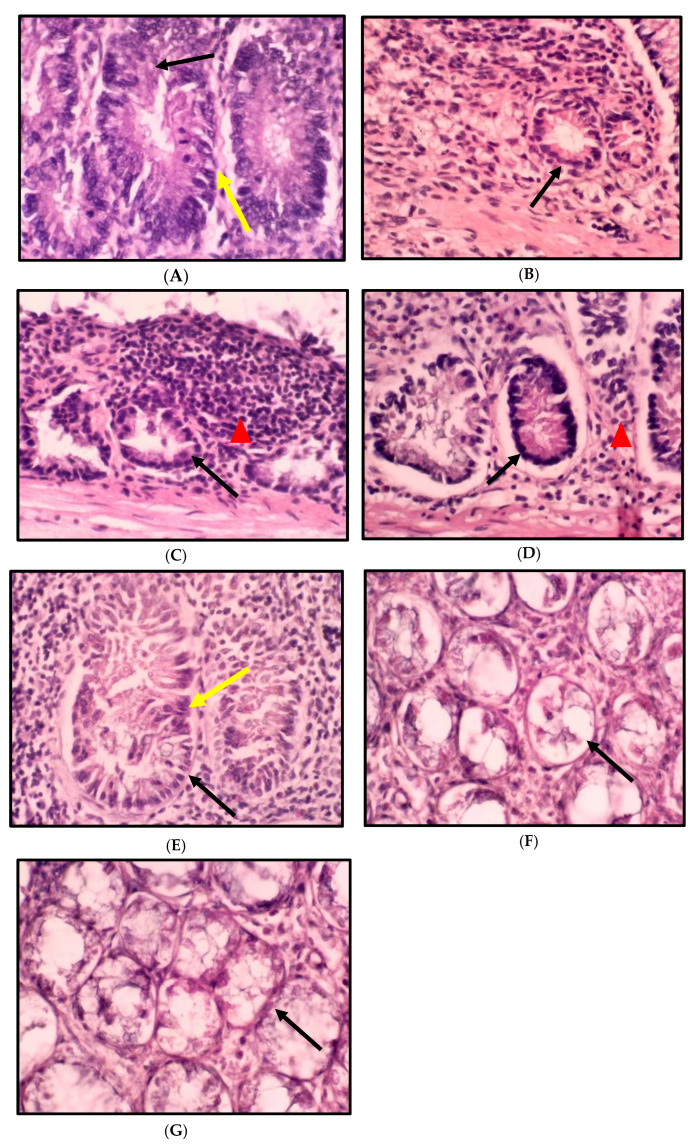
Cross-section of the rat colon stained with haematoxylin and eosin: (**A**) NC, normal diet + DMH; (**B**) H, high-fat diet; (**C**) HC, high-fat diet + DMH; (**D**) HCF, high-fat diet + DMH + 5-FU; (**E**) HCAP125, high-fat diet + DMH + 125 mg/kg *A. paniculata* ethanol extract; (**F**) HCAP250, high-fat diet + DMH + 250 mg/kg *A. paniculata* ethanol extract; (**G**) HCAP500, high-fat diet + DMH + 500 mg/kg *A. paniculata* ethanol extract. (Magnification, ×10.) For (**A**–**E**), the black arrow indicates aberrant crypt; the yellow arrow indicates larger, stratified, depolarized nuclei. Mucin and goblet cells diminish. Expanded crypts with slightly elongated nuclei are found in (**E**). (**F**,**G**) show a typical crypt without crowding, stratification, or mucin depletion. The red triangle indicates inflammatory cells in (**C**,**D**).

**Figure 4:**
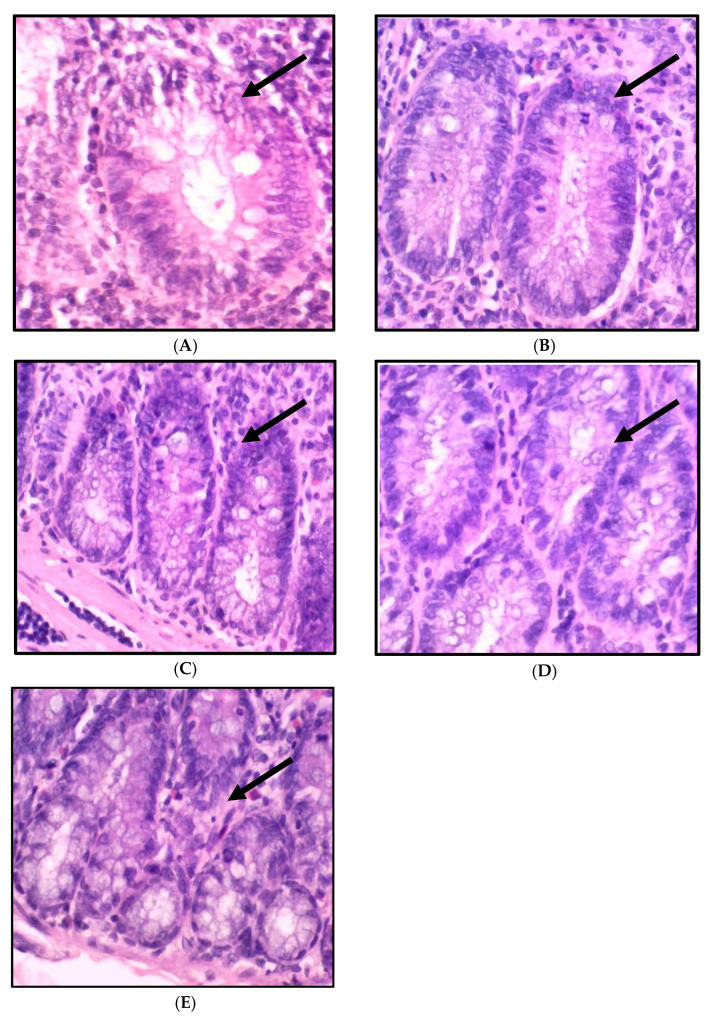
Histology classification of colon ACF types stained with haematoxylin and eosin (magnification, ×10): (**A**) one crypt, (**B**) two crypts, (**C**) three crypts, (**D**) four crypts, (**E**) and five or more crypts. The black arrow indicates the aberrant crypt foci.

The histopathological finding of colon tissue is shown in Figure 3 and Figure 4, which show the histology examination of the colon aberrant crypt foci (ACF) type using H&E staining. The histopathological changes in the colons of the rats consuming a standard and high-fat diet induced by DMH showed an abnormal crypt structure with elongated, enlarged, and stratified nuclei. A reduction in the number of goblet cells and mucin was also found in the colon tissue. In contrast, the colon morphology of the high-fat diet rats revealed a low grade of the abnormal crypt. The histology features of the colons in the treatment group HCAP125 showed slightly enlarged and elongated nuclei, while in the HCAP250 and HCAP500 groups, the colon was found to have a normal crypt with no crowding or stratification of nuclei and mucin depletion.

### 2.4. Impact of A. paniculata on the Overall Number of ACF in the Colon

The impact of *A. paniculata* on DMH and the high-fat-diet-treated ACF development in the rats’ colons are summarized in Table 3. The findings demonstrated that the rats fed with a normal diet did not develop ACF in their colons. Nevertheless, the only high-fat-diet-fed group (H) has ACF development without DMH induction. The DMH/HFD-treated group had significantly higher ACF development compared with the normal diet/DMH-treated group (*p* < 0.05). The primary effect analysis revealed that giving *A. paniculata* ethanolic extract and Fluorouracil significantly decreased the overall number of ACF contrasted with the DMH/HFD-treated rats; 500 mg/kg *A. paniculata* ethanol extract lowered the overall amount of ACF by 32%. A decrease of 19% of ACF was noticed in the treatment with 250 mg/kg *A. paniculata* ethanol extract, 16% in the treatment with 125 mg/kg *A. paniculata* ethanol extract, and 13% in the treatment with Fluorouracil. For the number of ACF consisting of one crypt, the treatment with 250 mg/kg *A. paniculata* ethanol extract significantly differed from the DMH/HFD-treated group. Contrasted with the DMH/HFD group, the treatment with 250 mg/kg *A. paniculata* ethanol extract generated significant results in ACF having four crypts, while treatment with 125 mg/kg *A. paniculata* produced substantial results in ACF having more than five crypts.

### 2.5. Impact of A. paniculata on the Retroperitoneal White Adipose Tissue (RpWAT)

**Figure 5:**
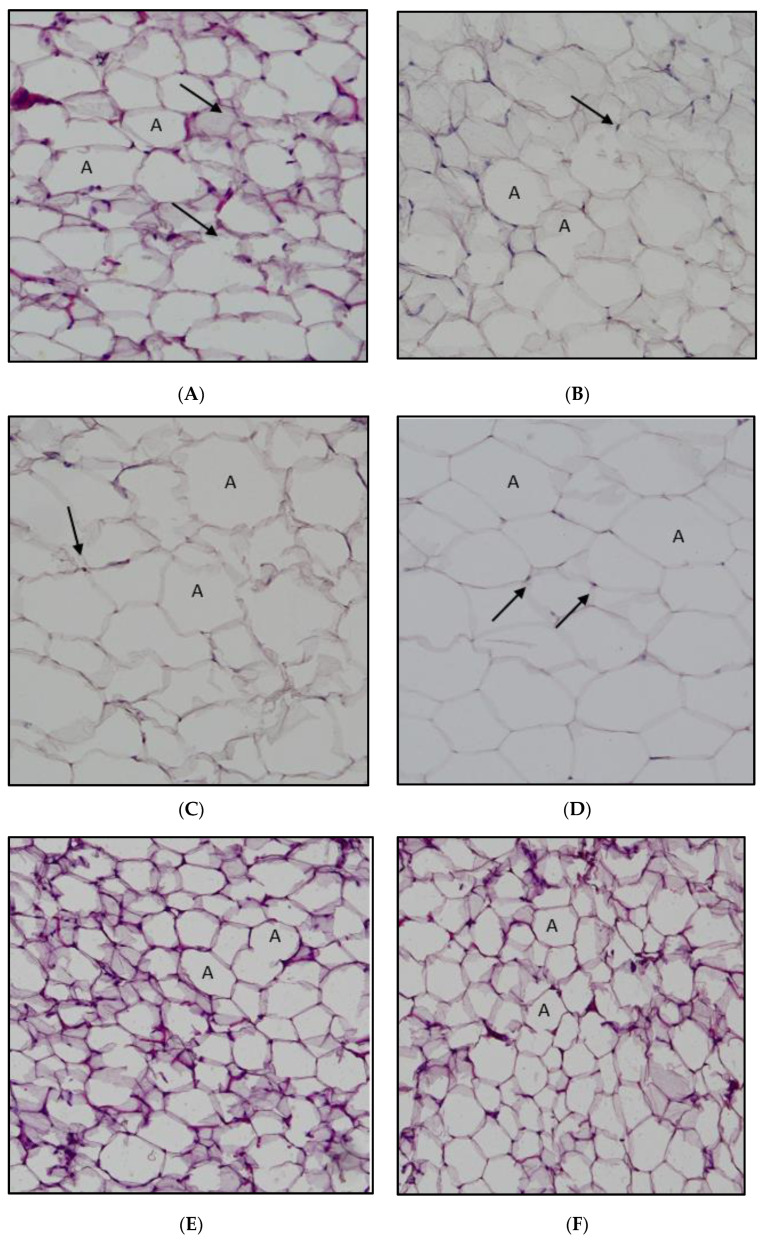

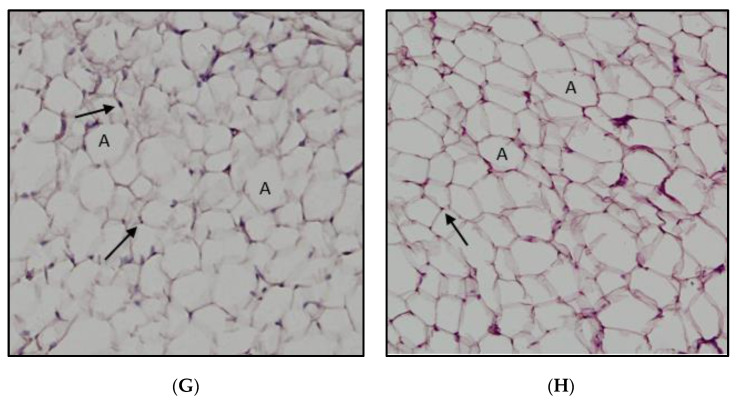
Cross-section of the rat adipose tissue stained with haematoxylin and eosin. (**A**) Normal diet group. (**B**) Normal diet + DMH group. (**C**) High-fat diet group. (**D**) High-fat diet + DMH group. (**E**) High-fat diet + DMH + 5-FU. (**F**) High-fat diet + DMH + 125 mg/kg *A. paniculata* ethanol extract. (**G**) High-fat diet + DMH + 250 mg/kg *A. paniculata* ethanol extract. (**H**) High-fat diet + DMH + 500 mg/kg *A. paniculata* ethanol extract group (10× magnification). The arrows indicate the nucleus, and “A” indicates adipocytes.

**Figure 6:**
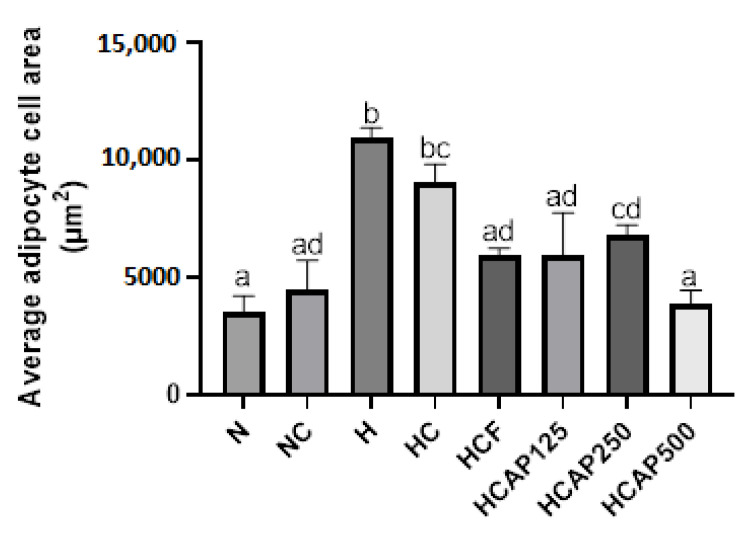
Impact of *A. paniculata* on the average adipocyte cell area of treated rats. The data are given as mean ± SE. Mean values with distinct alphabetical letters were significantly different at *p* < 0.05. (ANOVA, Tukey’s post hoc). N, normal diet; NC, normal diet + DMH; H, HFD; HC, HFD + DMH; HCF, HFD + DMH + 5-FU; HCAP125, HFD + DMH + 125 mg/kg *A. paniculata* ethanol extract; HCAP250, HFD + DMH + 250 mg/kg *A. paniculata* ethanol extract; HCAP500, HFD + DMH + 500 mg/kg *A. paniculata* ethanol extract.

The H&E staining of the retroperitoneal white adipose tissue is shown in Figure 5. The high-fat diet intake developed an expansion of adipocytes in the HFD group rats compared with the normal-diet-fed rats. Interestingly, *A. paniculata* ethanolic extract had the same effect as 5FU on reducing the size of the adipocytes. The adipocyte size (area) is demonstrated in Figure 6. The mean area of the high-fat diet rats and DMH/HFD rats was significantly larger than the normal chow diet rats and DMH/normal diet rats. The *A. paniculata* ethanolic extract in the dose of 125 mg/kg, 500 mg/kg, and the 5-fluorouracil groups reduced the mean adipocyte cell area significantly compared with the HFD/DMH group rats.

### 2.6. Impact of A. paniculata on the Serum Leptin, Adiponectin, and Insulin Concentration

**Figure 7:**
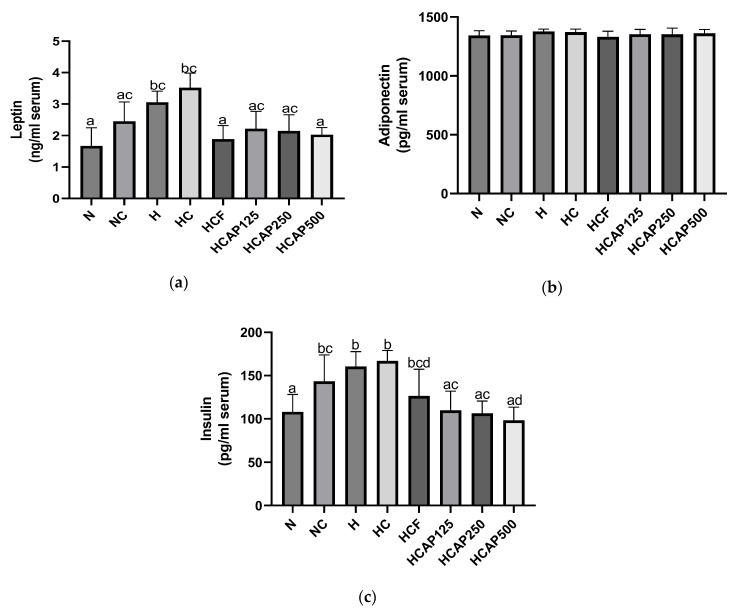
(**a**) Impact of *A. paniculata* on the serum leptin of treated rats. (**b**) Impact of *A. paniculata* on the serum adiponectin of treated rats. (**c**) Impact of *A. paniculata* on the serum insulin of treated rats. The data are given as mean ± SE. Mean values with distinct alphabetical letters were significantly different at *p* < 0.05 (ANOVA, Tukey’s post hoc). N, normal diet; NC, normal diet + DMH; H, HFD; HC, HFD + DMH; HCF, HFD + DMH + 5-FU; HCAP125, HFD + DMH + 125 mg/kg *A. paniculata* ethanol extract; HCAP250, HFD + DMH + 250 mg/kg *A. paniculata* ethanol extract; HCAP500, HFD + DMH + 500 mg/kg *A. paniculata* ethanol extract.

Figure 7a shows the impact of *A. paniculata* on serum leptin levels. The serum leptin level of the high-fat-diet-treated rats was significantly greater than the normal chow diet rats (*p* < 0.05). The administration of 5-FU and 500 mg/kg *A. paniculata* ethanolic extract significantly lowered the serum leptin level compared with DMH/HFD-induced rats. Figure 7b presents the effect of *A. paniculata* ethanol extract on serum adiponectin levels. *A. paniculata* ethanol extract did not significantly alter the serum adiponectin concentration. Figure 7c illustrates the impact of *A. paniculata* ethanol extract on serum insulin levels. A high-fat diet elevated the serum insulin level compared with the normal diet. The *A. paniculata* ethanol extract of all three doses significantly lowered the insulin level in the serum of the DMH/HFD-treated rats.

## 3. Discussion

Colorectal cancer (CRC) contributes to a higher mortality rate worldwide [19]. There are many risk factors for CRC. However, poor lifestyle and diet remain major risk factors for developing CRC. Recently, numerous studies on epidemiology have claimed that there is a solid link between high-fat diet intake and an elevated risk of CRC. Furthermore, many in vivo studies have illustrated that the chemically induced precancerous ACF formation increased due to high-fat diet consumption [20]. Therefore, in this experiment, the rats consumed a high-fat diet along with 1,2-dimethylhydrazine (DMH), which was induced to accelerate the CRC condition.

The typical treatment for CRC is surgical, followed by another approach, such as chemotherapy and immunotherapy, according to the disease onset [21]. Many chemopreventive studies have been conducted in the past, especially on the therapeutical plants [22]. Therefore, the existing research was intended to look at the potential chemopreventive impact of *A. paniculata* against DMH-induced colon cancer in Sprague Dawley rats under a high-fat diet condition.

*A. paniculata* is a herbaceous plant abundant in phytochemicals that help reduce the risk of getting cancer. These phytochemicals are proven to have antioxidant and chemopreventive agents [23,24]. The primary chemical components of *A. paniculata* are flavonoids, polyphenols, and terpenoids [25]. In this study, six main compounds were isolated from *A. paniculata* ethanol extract from terpenoids, flavonoids, and quinic acid groups. The primary diterpenoid compounds in *A. paniculata* are deoxyandrographolide, 14-deoxy-11,12-didehydroandrographide, isoandrographolide, and neoandrographolide [26]. However, the major flavonoids discovered in *A. paniculata* ethanol extraction are 5-hydroxy-7,8,2′,5′-tetramethoxyflavone, 5-hydroxy-7,8-dimethoxyflavone,5-hydrox-7,8,2′,3′tetramethoxyflavone, 2′-methyl ether, and 7-O-methylwogonin [27]. In a previous study, these terpenoids and flavanoids were exposed as stopping cancer proliferation by provoking apoptosis and cell cycle arrest [28,29,30]. This anti-cancer strategy occurs by activating the tumor suppressors p53 and p21, which inhibit the spread of cancer cells [31].

Moreover, these phytochemicals in ethanol extraction of the *A. paniculata* were discovered to slow down the oxidation of the cell due to their antioxidant properties. In a prior study, the potential of uptaking the free radical in *A. paniculata* extract was confirmed by NO, FRAP, and DPPH bioassays [32]. The anti-cancer potential of *A. paniculata* is directly attributed to the antioxidant activities that it possesses [33].

Weight loss is a common symptom faced by CRC patients. It happens due to cachexia caused by cancer. Cachexia is a condition described as losing body weight, primarily through losing weight in adipose tissue and skeletal muscle. Cachexia is brought on by a number of reasons, including decreased food intake, metabolic alterations, and inflammation [34]. This study observed body weight and RpWAT weight reduction in the DMH/HFD group despite no differences in total food intake. In the previous study, researchers faced the same body weight loss scenario in cancer-induced rats due to cachexia [35]. From the result, we speculate that the unexplainable weight loss of the rats might be due to cancer-associated cachexia.

The 1,2-dimethylhydrazine (DMH) is a procarcinogen used to cause aberrant crypt foci (ACF) in rats for CRC studies [36]. Nonetheless, the crypt development is not exclusively a result of DMH injection-induced inflammation. A high-fat diet can lead to obesity, which causes the adipocyte to release more adipokines that cause inflammation [20]. In this study, the histopathological examination of DMH/HFD rats demonstrated abnormal development in the colon tissue matching with previous findings [37]. Furthermore, DMH-induced rats with a high-fat diet had a considerably greater number of ACF than DMH-induced rats with a normal-fat diet. This result ties well with previous studies wherein Guang Ying et al. claimed that DMH-induced rats with a high-fat diet revealed a greater number of ACF than the moderate-fat-diet-treated rats [38]. This result again proves that a high-fat diet has a high-level potential to aggravate existing and initiate colorectal cancer [39]. *A. paniculata* ethanolic extract significantly improved the dysplastic state of the colon tissue and lowered the total number of ACF, suggesting the anti-cancer effect of *A. paniculata* against DMH/HFD-inducing rats. A similar result was proposed in the previous study, in which *A. paniculata* extract reduced the total number ACF and improved colon morphology in AOM-induced CRC rats [14].

With the progress of obesity, adipose tissue will endure tissue restoration in which the adipocyte increases in size and number. Hypertrophy of the adipocyte will lead to overloaded lipids, resulting in fluctuations in hormone secretion. The excess lipid will start to deposit in the organs, such as the pancreas, muscle, and liver [35]. In a recent study, the HFD affects the total area of the adipocyte, and the histological changes were significant. The average sizes of the adipocytes in high-fat diet rats and DMH/HFD rats were higher than those of the normal diet group and DMH/normal diet rats, respectively. Even in the histological examination, the adipocyte size of the HFD group and DMH/HFD group was more significant compared with normal diet and DMH/normal diet group rats. This again demonstrates agreement the previous study that found that HFD increased the adipocyte size [40]. The adipocyte size was reduced in DMH/HFD rats upon administering *A. paniculata* ethanol extract. This result agrees with that of Ramgopal Mopuri et al. According to their findings, *A. paniculata* extract may have beneficial effects in the fight against obesity and adipogenesis [41].

Adipocyte tissue secretes the adipokines leptin and adiponectin. Insulin resistance, hyperleptinemia, and diminished adipose-derived adiponectin secretion are associated with adipocyte tissue expansion [42]. Leptin is one of the obesity adipokines that accelerate the proliferation of CRC and also elevates insulin concentration [43,44]. However, adiponectin is negatively correlated with insulin resistance and obese [45,46]. Adiponectin is a type of adipokine that also acts as an anti-inflammatory and insulin-sensitizing agent [47]. In addition, the serum/plasma adiponectin concentration is also inversely related to CRC threat [48].

Previous animal research has demonstrated that leptin and insulin levels were high in rats fed with a high-fat diet, while there were no major differences in the adiponectin level [49]. In this study, the same theory was implied where due to the expansion of adipocytes, the leptin and insulin levels were elevated in the high-fat diet group compared with those in the normal diet group. However, the adiponectin levels were similar between groups. Interestingly, the leptin and insulin levels were regulated by the *A. paniculata* ethanol extract matching the study by Ding et al. [50]. From this perspective, we can conclude that leptin and adiponectin levels directly influence insulin levels in rats. The phytochemicals present in the *A. paniculata* were again shown to have the potential to regulate the adipokines associated with obesity and CRC [49].

## 4. Materials and Methods

### 4.1. Plant Material

The *A. paniculata* plant was purchased from Ethno resources Sdn Bhd, Sungai Buloh, Malaysia, and studied at the Herbarium Biodiversity Unit (UBD) at the Institute of Bioscience, University Putra Malaysia. The *A. paniculata* whole plant was oven dried and ground into powder. The 100 g of *A. paniculata* dried powder was soaked for 24 h in 1 L of 95% ethanol at normal temperature. The plant material underwent a maceration process three times for each batch to achieve the optimum yield for 3 days. The soaked extract was filtered using filter paper (Whatman, Maidstone, UK, 125 mm) to obtain the crude ethanolic extract. The solvent in the filtered ethanolic extract was evaporated under low pressure at 45 °C using a rotatory evaporator. The remaining solvent content was eliminated by oven drying (45 °C). The final ethanol crude extract was stored at −20 °C for further usage [14].

### 4.2. High-Fat Diet Preparation

The high-fat diet (HFD) had 414 calories per 100 g and consisted of 17% protein, 40% fat, and 43% carbohydrates; the diet contained 6% ghee (Crispo, CrispoTato (M) Sdn Bhd, Kuala Lumpur, Malaysia), 6% corn oil (Vecorn, Yee Lee Corporation Berhad, Kuala Lumpur, Malaysia), 68% standard chow pellet (Gold Coin Feedmills (M) Sdn Bhd, Selangor, Malaysia), and 20% milk powder (Dutch Lady, Dutch Lady Milk Industries Berhad, Selangor, Malaysia). A normal chow pellet has 306.2 calories per 100 grams of protein, 3% fat, and 48.8% carbohydrates [51]. The high-fat diet was freshly made and refrigerated weekly.

### 4.3. Animal Study

In the Animal House, Faculty of Medicine and Health Sciences, University Putra Malaysia, 48 healthy male Sprague Dawley (SD) rats (150–200 g) were placed in separate cages. The Animal Care and Use Committee of University Putra Malaysia approved the study. One week was spent acclimating these rats to the normal chow diet and water. After acclimation, the rats were split into eight groups. Each group had six rats.

Group N: Standard chow diet + tap water.

Group NC: Standard chow diet + DMH.

Group H: High-fat diet + tap water.

Group HC: High-fat diet + DMH.

Group HCF: High-fat diet + DMH + Fluorouracil

Group HCAP125: High-fat diet + DMH + 125 mg/kg *A. paniculata* ethanol extract.

Group HCAP250: High-fat diet + DMH + 250 mg/kg *A. paniculata* ethanol extract.

Group HCAP500: High-fat diet + DMH + 500 mg/kg *A. paniculata* ethanol extract.

N indicates normal, C indicates cancer, H indicates high-fat diet, F indicates fluorouracil, and AP indicates *A. paniculata*.

The rats were orally administered a standard chow and high-fat diet for 20 consecutive weeks; 40 mg/kg DMH was injected subcutaneously at the groin region once a week for 10 weeks [19], and 35 mg/kg of fluorouracil (5-FU) was given by intra-peritoneal injection two times a week for 20 weeks. Three different doses of *A. paniculata* ethanol extract were orally administrated daily for 20 weeks. Every week till the last week of the in vivo experiment, the food intake and body weight were measured [14].

### 4.4. Chemicals

The 1,2-dimethylhydrazine was diluted in 1 mM ethylenediaminetetraacetic acid (EDTA, Sigma Co., Ronkonkoma, NY, USA), and 10% sodium hydroxide was used to change the pH to 6.5. To cause cancer, it was given subcutaneously at a dose of 40 mg/kg once every week for ten weeks [19]; 2 g of 5-Fluorouracil was diluted in 100 mL of 0.9% normal saline to achieve a final concentration of approximately 2000 mg/100 mL (20 mg/mL) and then given intraperitoneally at a dosage of 35 mg/kg twice weekly for 20 weeks [14].

### 4.5. Phytochemical Screening

UHPLC was used to separate the chemical components (ACQUITY UPLC I-Class system from Waters). The separation was carried out with a 40 °C ACQUITY UPLC HSS T3 column (100 mm × 2.1 mm × 1.8 m). Gradient elution of 0.1% formic acid-containing water (A) and acetonitrile (B) was as follows: 0% B; 0.5% B; 16:00 B; 18:00 B; 20% B. One liter was injected at 0.6 mL/min. The UHPLC system was linked to a Waters Vion IMS QTOF hybrid mass spectrometer. The source temperature was 120 °C, the desolvation gas temperature was 550 °C, the desolvation gas flow was 800 L/h, and the cone gas flow was 50 L/h. Nitrogen (>99.5%) was used as the desolvation and cone gas. The HDMSE data were gathered at 0.1 s/scan from 50 to 1500 *m*/*z*. During the run, two scans with different collision energies (CE) were alternately acquired: a low-energy (LE) scan with a constant CE of 4 eV and a high-energy (HE) scan with a ramp from 10 to 40 eV. Collision-induced dissociation (CID) gas was utilized, which was 99.999% pure argon [52].

### 4.6. Biochemical Test

The rats’ blood samples were stored in a plain serum tube by puncturing their hearts. For 10 min, the plain tubes were spun at 3000 rpm. For further investigation, the serum was frozen at −80 °C. ELISA kits from Elabscience were used to measure the concentrations of leptin, adiponectin, and insulin in the blood [53].

### 4.7. Histopathological Examination

During the rat sacrifice, 10% formalin was used to fix the colon and adipose tissues for 24 h. After fixation, the colon and adipose tissue were placed in a cassette. The specimen cassettes underwent tissue processing. Next, the cassettes were fixed in paraffin wax and sectioned using a rotatory microtome with a 5 µm thickness. The sliced specimen was placed on slides. The specimen slides were stained with hematoxylin and eosin. The stained specimen slides were covered using slip slides [37]. The histopathology changes were examined by a pathologist.

### 4.8. ACF Counting

The stained colon segments were placed on an electric light microscope, and the number of crypt foci was quantified manually on random observation fields at 4× and 10× magnification [54].

### 4.9. Adipocyte Area Counting

The stained adipocyte histology pictures were captured using an electric light microscope at 10× magnification. The image was then uploaded into ImageJ for area counting. A total of 100 individual adipocytes were calculated for each slide manually by using the ImageJ application [55].

### 4.10. Statistical Analysis

The data from the food intake, body weight, organ weight, biochemical test, and adipocyte count were collected. Each experiment was repeated a minimum of three times. Independent samples were evaluated with a one-way ANOVA and subsequently Tukey’s post hoc test using IBM SPSS statistics version 27. *p* < 0.05 was significant, and all the values were given as the mean ± SE [39].

## 5. Conclusions

In a nutshell, these findings indicate that HFD worsened the condition of ACF induced by DMH. This happened due to the imbalanced ratio between leptin and adiponectin. Therefore, *A. paniculata* played an essential role in this study as a potential intervention for colorectal cancer. The anti-cancer-rich phytochemicals such as 12S-Hydroxyandrographolide and Genistein-7,4′-di-O-β-D-glucoside present in the *A. paniculata* ethanol extract aided in altering the morphological identity of the crypt from abnormal to almost normal and reduced the total number of ACF. Moreover, *A. paniculata* extract too interfered in the expansion of the adipocyte, which helped regulate the serum levels of leptin and insulin. This could be recognized as the anti-adipogenic and anti-obesity properties of the *A. paniculata* extract. Overall, *A. paniculata* shows potential chemopreventive effects on CRC, and along with that, it helps to regulate the obesity factors such as adipokine that aggravate the proliferation of CRC.

## Figures and Tables

**Table 1:** Identification of primary phytochemical component of *A. paniculata* ethanol extract by UHPLC-QTOF-MS.

Compounds	Molecular Formula	RT (min)	Molecular Mass	Observed (*m*/*z*)	Mass Error (ppm)
19β-Glucosyl-14-deoxy-11,12-didehydroand-rographoside	C_20_H_28_O_4_	12.6	332.1983	331.191	−1.3
3,4-O-Dicaffeoylquinic acid	C_25_H_24_O_12_	10.65	516.1273	515.12	1
Genistein-7,4′-di-O-β-D-glucoside	C_27_H_30_O_15_	9.12	594.1594	593.1521	1.6
12S-Hydroxyandrographolide	C_20_H_32_O_6_	10.73	368.2198	367.2125	−0.3
10-Hydroxyligustroside	C_25_H_32_O_13_	12.98	540.1845	539.1772	0.3
19β-Glucosyl-14-deoxyandrographoside	C_26_H_40_O_9_	13.47	496.2672	495.26	0

RT stands for “retention time”.

**Table 2:** Impact of *A. paniculata* ethanolic extract on food intake, body weight, RpWAT, and colon weight and colon length.

Group	Total Food Intake (kJ)	Body Weight (g)		RpWAT Weight		Colon Weight		Colon Length (cm)
		Initial	Final	(g)	(%BW)	(g)	(%BW)	
N	7477.40 ± 873.70 ^a^	227.50 ± 10.9	440.33 ± 12.01 ^a^	6.48 ± 0.71 ^a^	1.47	1.48 ± 0.22 ^a^	0.34	17.53 ± 0.58 ^a^
NC	7917.40 ± 1515.19 ^a^	226.50 ± 6.4	448.17 ± 16.39 ^a^	9.16 ± 1.60 ^ab^	2.00	1.26 ± 0.10 ^a^	0.28	14.08 ± 0.68 ^a^
H	12342.26 ± 237.56 ^b^	240.00 ± 5.6	571.83 ± 20.20 ^b^	22.85 ± 3.52 ^c^	3.94	1.67 ± 0.22 ^a^	0.30	15.83 ± 0.94 ^a^
HC	12319.72 ± 991.74 ^b^	202.20 ± 19.8	453.33 ± 19.13 ^a^	9.17 ± 2.54 ^ab^	1.95	1.43 ± 0.12 ^a^	0.32	16.07 ± 1.37 ^a^
HCF	12586.98 ± 502.32 ^b^	239.2 ± 7.1	461.17 ± 6.09 ^a^	13.07 ± 3.18 ^ac^	2.83	1.64 ± 0.23 ^a^	0.35	16.07 ± 1.37 ^a^
HCAP125	12422.76 ± 705.18 ^b^	238.30 ± 3.3	503.67 ± 18.07 ^ab^	10.90 ± 1.56 ^ac^	2.13	1.70 ± 0.13 ^a^	0.34	15.75 ± 1.11 ^a^
HCAP250	12525.80 ± 75.72 ^b^	247.50 ± 8.8	512.50 ± 24.24 ^ab^	14.83 ± 4.32 ^bc^	2.81	1.54 ± 0.11 ^a^	0.30	15.30 ± 0.80 ^a^
HCAP500	12477.50 ± 271.30 ^b^	235.8 ± 6.9	474.17 ± 7.45 ^a^	7.72 ± 1.07 ^ab^	1.64	1.70 ± 0.22 ^a^	0.36	16.50 ± 1.65 ^a^

The data are given as mean ± SE. Mean values with the distinct alphabets in the similar column were significantly different at *p* < 0.05 (ANOVA, Tukey’s post hoc). N, normal diet; NC, normal diet + DMH; H, HFD; HC, HFD + DMH; HCF, HFD + DMH + 5-FU; HCAP125, HFD + DMH + 125 mg/kg *A. paniculata* ethanol extract; HCAP250, HFD + DMH + 250 mg/kg *A. paniculata* ethanol extract; HCAP500, HFD + DMH + 500 mg/kg *A. paniculata* ethanol extract. RpWAT, retroperitoneal white adipose tissue; HFD, high-fat diet.

**Table 3:** Impact of *A. paniculata* on the overall number of ACF in the colon.

Group	No. of Crypts per ACF					Total No. of ACF/Colon
	One Crypt	Two Crypts	Three Crypts	Four Crypts	Five or More Crypts	
N	0	0	0	0	0	0
NC	5.25 ± 1.60 ^ab^	3.50 ± 0.90 ^a^	1.50 ± 0.70 ^a^	1.62 ± 0.56 ^ab^	3.25 ± 1.08 ^a^	15.37 ± 0.26 ^ab^
H	1.37 ± 0.73 ^b^	2.00 ± 0.94 ^a^	3.50 ± 0.56 ^a^	2.25 ± 0.45 ^ab^	6.12 ± 1.67 ^ab^	15.12 ± 0.12 ^ab^
HC	7.50 ± 1.42 ^a^	3.87 ± 0.63 ^a^	1.62 ± 0.46 ^a^	0.87 ± 0.35 ^b^	6.50 ± 1.50 ^ab^	20.37 ± 0.80 ^c^
HCF	5.87 ± 1.41 ^ab^	1.87 ± 0.63 ^a^	2.00 ± 0.82 ^a^	0.37 ± 0.18 ^b^	7.25 ± 1.94 ^ab^	17.62 ± 0.37 ^d^
HCAP125	2.80 ± 1.15 ^ab^	1.80 ± 0.58 ^a^	1.00 ± 0.31 ^a^	0.60 ± 0.40 ^b^	10.80 ± 0.86 ^b^	17.00 ± 0.83 ^ad^
HCAP250	1.16 ± 0.47 ^b^	2.66 ± 0.71 ^a^	3.83 ± 0.70 ^a^	3.50 ± 0.76 ^a^	5.16 ± 0.83 ^ab^	16.33 ± 0.33 ^abd^
HCAP500	5.00 ± 1.69 ^ab^	1.66 ± 0.33 ^a^	1.66 ± 0.33 ^a^	1.16 ± 0.98 ^ab^	4.33 ± 1.72 ^ab^	13.83 ± 0.87 ^b^

The data are given as mean ± SE. Mean values with distinct alphabetical letters in the same column were significantly different at *p* < 0.05 (ANOVA, Tukey’s post hoc). N, normal diet; NC, normal diet + DMH; H, HFD; HC, HFD + DMH; HCF, HFD + DMH + 5-FU; HCAP125, HFD + DMH + 125 mg/kg *A. paniculata* ethanol extract; HCAP250, HFD + DMH + 250 mg/kg *A. paniculata* ethanol extract; HCAP500, HFD + DMH + 500 mg/kg *A. paniculata* ethanol extract.

## Data Availability

Not applicable.
